# Does Depression and Anxiety Mediate the Relation between Limited Health Literacy and Diet Non-Adherence?

**DOI:** 10.3390/ijerph17217913

**Published:** 2020-10-28

**Authors:** Ivana Skoumalova, Andrea Madarasova Geckova, Jaroslav Rosenberger, Maria Majernikova, Peter Kolarcik, Daniel Klein, Andrea F. de Winter, Jitse P. van Dijk, Sijmen A. Reijneveld

**Affiliations:** 1 Department of Health Psychology and Research Methodology, Faculty of Medicine, P.J. Safarik University, Trieda SNP 1, 040 01 Kosice, Slovakia; andrea.geckova@upjs.sk (A.M.G.); Jaroslav.rosenberger@upjs.sk (J.R.); peter.kolarcik@upjs.sk (P.K.); 2 Graduate School Kosice Institute for Society and Health, Faculty of Medicine, P.J. Safarik University in Kosice, Trieda SNP 1, 040 01 Kosice, Slovakia; j.p.van.dijk@umcg.nl; 3 Department of Community & Occupational Medicine, University Medical Center Groningen, University of Groningen, Antonius Deusinglaan 1, 9713 AV Groningen, The Netherlands; a.f.de.winter@umcg.nl (A.F.d.W.); s.a.reijneveld@umcg.nl (S.A.R.); 4 Olomouc University Social Health Institute, Palacky University, Univerzitni 22, 771 11 Olomouc, Czech Republic; 5 FMC-Dialysis Services Slovakia, Trieda SNP 1, 040 01 Kosice, Slovakia; mamajern@gmail.com; 6 2nd Department of Internal Medicine, P.J. Safarik University, Trieda SNP 1, 040 01 Kosice, Slovakia; 7 Institute of Mathematics, Faculty of Science, P. J. Safarik University, Jesenna 5, 040 01 Kosice, Slovakia; daniel.klein@upjs.sk

**Keywords:** health literacy, dialyzed patients, depression, anxiety, diet non-adherence, stage 5 CKD

## Abstract

Limited health literacy (HL), depression and anxiety are common in dialyzed patients and affect health outcomes and self-management. We explored whether depression and anxiety mediate the association of HL with diet non-adherence (DN-A) in dialyzed patients. We performed a cross-sectional study in 20 dialysis clinics in Slovakia (*n* = 452; mean age: 63.6 years; males: 60.7%). Hierarchical cluster analysis was performed to create three HL groups. Logistic regression adjusted for age, gender and education was used to explore whether depression and anxiety mediate the association of HL with DN-A. Patients in the moderate HL group were more likely to be non-adherent to diet (OR (Odds Ratio)/95% CI: 2.19/1.21–3.99) than patients in the high HL group. Patients in the low HL and moderate HL group more likely reported depression or anxiety. Patients reporting depression (OR/95% CI: 1.94/1.26–2.98) or anxiety (OR/95% CI: 1.81/1.22–2.69) were more likely to be non-adherent with diet. Adjustment for depression reduced the association between moderate HL and DN-A by 19.5%. Adjustment for anxiety reduced the association between moderate HL and DN-A by 11.8%. Anxiety and depression partly mediated the association of HL with DN-A. More attention should be paid to treating patients’ psychological distress to ensure adequate adherence with recommended diet.

## 1. Introduction

Chronic kidney disease (CKD) is a rapidly increasing public health problem with a global prevalence of 8–16% [1,2]. CKD represents a great burden for the economy as well as for the health care system. Thus far, we have not succeeded in reversing the increasing prevalence of CKD nor its progression to more severe stages. Most of this burden is due to the final stage of CKD (stage 5 CKD). Stage 5 CKD considerably affects patients’ lives; it decreases physical functioning, affects mental health and social life [3] and thus represents a great challenge for public health policies and healthcare.

Effective treatment of stage 5 CKD in patients undergoing hemodialysis requires adequate diet adherence but may be hampered or facilitated by patients’ health literacy (HL) limitations and capacities. HL is defined as knowledge, motivation, and competence of patients to access, understand, appraise, and apply health information in order to make judgments and decisions in their everyday life concerning healthcare, disease prevention, and health promotion to maintain or improve their quality of life [4]. Limited HL is associated with adverse health outcomes such as more hospitalizations and cardiovascular events [5], higher mortality [6,7] and worse health related self-management, e.g., missed dialysis sessions [5] and non-adherence to diet and fluid intake recommendations [8]. Increasing a patient’s HL is also considered as a cost-effective intervention area to promote and maintain patients’ health [9,10].

Depression and anxiety are common in dialyzed patients [11,12,13,14]. The prevalence ranges from about 12 to 52% for anxiety and from about 23 to 42% for depression [15,16,17], among others depending on the assessment tools used. Depression and anxiety are both associated with impaired quality of life in dialyzed patients [18,19,20], and with worse health outcomes and increased mortality [21,22,23,24,25,26,27,28]. Furthermore, both are associated with withdrawal from dialysis [25,29], and with diet non-adherence [30,31]. Both anxiety and depression might thus hamper self-management behavior and cooperative engagement with health care providers needed for effective treatment and maintaining the quality of life of patients in this stage of the disease [12,32].

Research has shown a link of limited HL and non-adherence as well as the link of depression and anxiety and non-adherence, but their joint effects are still not fully clear and neither are the underlying mechanisms [33,34]. Patients with limited HL may have problems navigating the disease in its complexity and coping with its burden, which might result in increased levels of depression and anxiety. It is confirmed by research [32,34,35] that limited HL is associated with increased levels of depression and anxiety in dialyzed patients. Increased levels of depression and/or anxiety may affect diet adherence. HL may, therefore, have an indirect effect on diet non-adherence via depression and/or anxiety. As evidence on this mechanism is lacking, our aim was to assess the relation of HL, depression and anxiety with diet non-adherence and to assess whether anxiety and depression mediate the association of HL and diet non-adherence in hemodialyzed patients.

## 2. Materials and Methods

### 2.1. Sample and Procedure

The data were collected within a network of 20 dialysis clinics in Slovakia from January 2018 to November 2018. These dialysis clinics belong to the private dialysis network—Fresenius Medical Care-dialysis services in Slovakia and cover about 20% of the total Slovak dialysis population. We included patients aged over 18 years, with the diagnosis of stage 5 CKD and undergoing hemodialysis for at least 3 months. We excluded those who were not able to fill in the questionnaire (due to dementia or mental retardation, inability to read the Slovak language) and those who had an acute severe intercurrent illness, according to the medical records.

The patients were approached during their routine visit at the dialysis center. Those who agreed to participate in the study signed an informed consent and filled in questionnaires. We used the tablets with the data recorded on the online platform. Full confidentiality was assured with the personal identification code.

### 2.2. Ethics

The study was approved by the relevant ethics committees: the Ethics Committee of the Faculty of Medicine P.J. Safarik University (15N/2017) and the Ethics Committee of FMC-dialysis services.

### 2.3. Measures

Diet non-adherence was measured using a questionnaire. Respondents were asked: “How often do you break diet recommendations? Breaking diet recommendations means eating foods which are not recommended, respectively prohibited by the medical personnel.” with the 6 answers offered: (1) do not break; (2) once a month or less; (3) once per 2–3 weeks; (4) once a week; (5) 2–3 times a week; (6) always. For further statistical analyses, we dichotomized this variable as once a week or more vs. less to non-adherent with the diet vs. adherent, based on the disease specific guidelines [36], other research concerning adherence [37] and renal experts advise.

Health literacy (HL) was measured using the Slovak version of the Health Literacy Questionnaire (HLQ) [38]. It is a multidimensional tool for measuring nine domains of HL [39] (see Appendix A). These domains are related to accessing, understanding and using health information to manage one’s health. The questionnaire consists of nine, highly reliable domains of HL (Cronbach’s Alpha in our sample—0.90). There is no overall total score for this questionnaire; higher mean score in a particular domain indicates a better HL in that domain [39]. For further analyses of HL, we used the expectation maximization algorithm (EMA) [40] to replace missing HLQ scores. The EMA replaces missing scores per domain for each respondent. The condition, that a maximum of two values were missing for the domains with the 4–5 items and the maximum of three values are missing for the domain with the 6 items, had to be met [41]. We excluded respondents who had more missing values in a particular domain.

**Figure 1:**
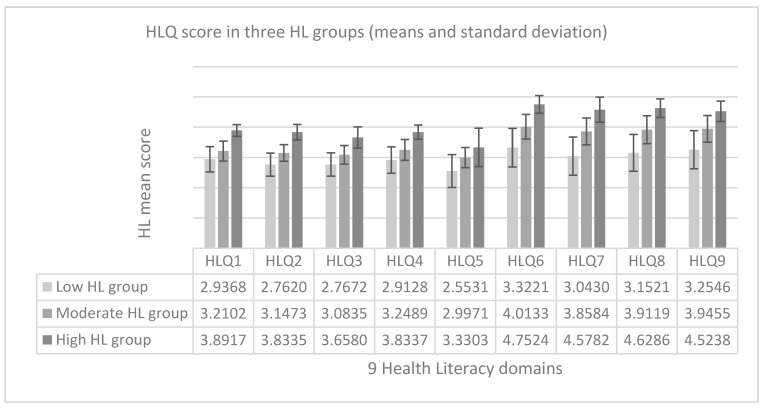
Health Literacy Questionnaire (HLQ) mean scores of 9 domains in three health literacy (HL) groups.

Next, we categorized this measure using hierarchical cluster analysis [42] on standardized z-scores of all HL domains. We created clusters of cases with similar HL characteristics. This method minimizes within-cluster variance in a stepwise manner leading to clusters that are as different as possible. Three clusters were used for further analyses (low HL group, moderate HL group, high HL group), representing different levels of HL consistently across all domains in a particular cluster. HLQ mean scores of 9 domains in three HL groups of patients are described in Figure 1.

Depression and anxiety were measured by the Hospital Anxiety and Depression Scale (HADS) [43,44,45], a 14-item questionnaire assessing depression (seven items; Cronbach’s Alpha—0.75) and anxiety (seven items; Cronbach’s Alpha—0.81) symptoms. The respondent was asked how he/she has been feeling in the past week. A Likert scale from 0 to 3 was used to assess the degree of the severity of the symptoms of depression and anxiety, leading to a total score for each subscale ranging from 0 to 21. Clinically, a score of 0–7 is considered as normal, 8–10 as borderline level, 11–21 as an abnormal level of depression/anxiety. We dichotomized this variable as no/low symptoms (<8) vs. moderate/severe symptoms (>8) of depression and anxiety [11,46,47,48].

Socio-demographic data (age, gender and education (lower: elementary and apprenticeship vs. higher: secondary and university)) were measured using the questionnaire.

### 2.4. Statistical Analyses

First, we assessed the socio-demographic characteristics, depression, anxiety and diet non-adherence of the sample. Second, we assessed the association between HL clusters (used as a categorical variable) with depression and anxiety (dichotomized) using logistic regression models adjusted for age (continuous) gender and education. Thirdly, we assessed the associations between HL clusters, depression and anxiety with diet non-adherence (yes/no), using logistic regression models adjusted for age, gender and education. We reported the odds ratio (OR) with 95% confidence interval (CI). Model 1 tested the association of HL groups, depression and anxiety each separately with diet non-adherence. Model 2 was adjusted separately for depression and anxiety. The degree of reduction in the ORs was computed using the formula: (OR (crude)—OR (adjusted)/(OR (crude)—1) × 100%. The listwise deletion of missing data was used in all logistic regression models. All statistical analyses were performed using SPSS v. 23.0 for Windows (IBM SPSS, 2015, Armonk, NY, USA).

## 3. Results

### 3.1. Baseline Characteristics

We included 567 dialyzed patients (70.1% of those approached); 25 patients were excluded due to not filling in the questionnaire related to HL properly, leading to a sample of 542 patients included in cluster analysis. In the next step, we excluded respondents with missing data on explored variables (*n* = 63) resulting in the final sample of 479 patients (mean age 63.6 years (standard deviation: 14.1 years). The time on dialysis in our sample ranged from 3 months to 36 years (mean = 5.3 years). The majority of our sample had low HL (31.5%) or moderate HL (55.3%). The patient group with high HL covered 13.2% of patients. Patients frequently reported depression (26.9%) and anxiety (32.2%). Non-adherence to diet recommendations was reported by 43.0% of patients (Table 1).

### 3.2. Associations of Health Literacy with Depression and Anxiety

We found that patients in the low HL group and moderate HL group were more likely to have moderate/severe symptoms of depression (low HL group OR 8.06; 95% CI: 2.74–23.70/moderate HL group OR 5.16; 95% CI: 1.78–14.91) and anxiety (low HL group OR 3.01; 95% CI: 1.45–6.27/moderate HL group OR 2.21; 95% CI: 1.09–4.46). Findings are described in Table 2.

### 3.3. Associations of Health Literacy, Depression and Anxiety with Diet Non-Adherence

We found that patients in the moderate HL group were more likely to be non-adherent to diet (OR 2.19; 95% CI: 1.21–3.99) than patients in the high HL group (Model 1, Table 3). Moreover, those patients with moderate/severe symptoms of depression (OR 1.94; 95% CI: 1.26–2.98) and moderate/severe symptoms of anxiety (OR 1.81; 95% CI: 1.22–2.69) were more likely to be non-adherent with diet (Model 1, Table 3).

### 3.4. Mediation Effect of Depression and Anxiety on the Relation of Hl with Diet Non-Adherence

Adjustment for depression and anxiety separately reduced the association between HL and diet non-adherence (Model 2, Table 3). Adjustment for depression reduced the association between moderate HL and diet non-adherence by 19.5%. Adjustment for anxiety reduced the association between moderate HL and diet non-adherence by 11.8%.

## 4. Discussion

In this cross-sectional study among dialyzed patients, we assessed the association of HL with diet non-adherence and the pathway through which depression and anxiety contribute to this association as mediators. We found that patients in the moderate HL group were more likely to be non-adherent with diet, but we did not confirm it in patients in the low HL group. This is in line with the research findings of Stømer et al. [49] who focused on HL of CKD patients and its association with adherence to lifestyle recommendations and brought similar findings to ours, that patients with moderate HL reported worse adherence than patients with higher HL, but they did not find low HL group to be significantly different than moderate or high HL group. As end stage renal disease (ESRD) is a result of long-term health problems and occurred frequently in the elderly, patients with low HL may already depend their diet adherence on caregivers (spouse, family members), or simplify their diet regime to avoid non-adherence. Another explanation might be that patients with low HL do not feel competent enough to question the healthcare providers’ opinions and recommendations, they do not search for other sources of information on diet and thus are more likely to adhere to the recommendations they obtain from health care providers within the dialysis care. On the contrary, research of Qobadi et al. [32] focused on HL in ESRD patients and its association with self-care behavior and found it to be worse in patients with inadequate HL than in patients with marginal or adequate HL. An explanation might be the use of a different measurement tool (S-TOFHLA) focusing on functional HL (reading and numeracy skills). Nevertheless, decreased HL capacities in finding, understanding, and acting upon relevant health information from health care providers or other sources of health information related to the disease apparently represent a barrier for adequate adherence.

We found that depression, as well as anxiety, partly mediated the association between lower HL and diet non-adherence. Qobadi et al. [32] also confirmed that depression and anxiety mediated the relation of HL and self-care in ESRD patients. The mediating role of depression and anxiety on the association of HL and adherence was confirmed also in other patient groups, as was shown by Lin et al. [50], who found that eHealth literacy had a direct and indirect effect, through psychological distress—depression and anxiety, on medication adherence in older patients with heart failure. In our sample, patients with lower HL were more likely to be depressed and anxious and this corresponds with the findings of Dodson et al. [34] and Qobadi et al. [32] who found that dialyzed patients with lower HL reported more symptoms of depression and anxiety than patients with higher HL. Moreover, those patients in our sample with moderate/severe symptoms of depression and anxiety were more likely to be non-adherent. The association of depression and anxiety with non-adherence was confirmed also by other research studies [30,31]. We interpret this as that increased levels of depression and anxiety in patients with limited HL may prevent them to use their, however limited, capacities to find, understand and act upon health information, which leads to the less effective management of their diet and to the decreased ability to adhere to diet recommendations. This may be explained also by decreased ability to communicate problems related to diet with relevant health care providers due to feelings of failure, passivity or feelings of uncertainty and thus to continue to eat foods that are not recommended.

### 4.1. Strengths and Limitations

The main strength of our study is the use of a representative sample covering approximately 20% of all dialyzed patients in Slovakia. We also used a set of instruments for measuring HL and depression and anxiety with good reliability. In addition, our study provides important information on the topic of psychological distress represented by the symptoms of depression and anxiety in dialyzed patients, which was not sufficiently studied to this date in relation to HL. The major limitation of our study is that we had a cross-sectional design which limits causal inferences, especially regarding mediation analysis. Another limitation is that we could not incorporate the information on the history of psychological diagnosis of depression and/or anxiety in our analyses, to address pathways.

### 4.2. Implications

Our findings on the mediating role of depression and anxiety in relation to HL and diet non-adherence imply that attention is needed to focus on early and regular screening for the symptoms of depression and anxiety and their treatment in dialyzed patients. Decreased capacities in HL may lead to the feelings of failure, frustration, passivity or uncertainty in relation to treatment and health improvement and thus result in decreased diet adherence. Feelings of confusion related to health information and treatment in connection to anxiety or depression may affect communication with health care providers, thus they should pay more attention to encourage patients to be active in participation and offer psychological support where needed.

Future research should aim for a deeper understanding of the pathways of the relation of HL and negative psychological status in dialyzed patients which affects their ability to adhere to prescribed treatment. Qualitative studies on this topic might help to identify other factors associated with psychological distress in dialyzed patients. Moreover, interventions are needed to combat depression and anxiety in dialyzed patients and thus maintain their health, quality of life, adequate cooperation with health care providers and adherence to treatment. Finally, experimental studies are required on whether interventions targeting depression and anxiety in dialyzed patients indeed increase levels of HL capacities and so improve diet adherence.

## 5. Conclusions

We found that patients with moderate HL were more likely to be non-adherent with diet; the same is true for patients who had moderate/severe symptoms of depression and/or anxiety. Depression, as well as anxiety, partly mediated the association between HL and diet non-adherence. The findings of our study contribute to the knowledge of the importance of increasing HL in dialyzed patients with the aim to improve their adherence to diet recommendations and of the importance of focusing on the role of depression and anxiety in the relation of HL and non-adherence. Considering both aspects (HL and psychological distress represented by present symptoms of depression and/or anxiety) may help to improve the overall health and quality of life of dialyzed patients.

## Figures and Tables

**Table 1:** Characteristics of the total sample, gender, education, health literacy (HL), depression, anxiety and diet non-adherence (*n* = 479).

Characteristics	Total Sample *n* (%)
Gender	
male	287 (59.9)
female	192 (40.1)
Education	
lower	237 (49.5)
higher	242 (50.5)
Health literacy	
low HL group	151 (31.5)
moderate HL group	265 (55.3)
high HL group	63 (13.2)
Depression	
no symptoms	350 (73.1)
moderate/severe symptoms	129 (26.9)
Anxiety	
no symptoms	325 (67.8)
moderate/severe symptoms	154 (32.2)
Diet adherence	
adherent	273 (57.0)
non-adherent	206 (43.0)

**Table 2:** The association of HL with depression and anxiety adjusted for age and gender (*n* = 479).

Health Literacy	Depression OR (95% CI)	Anxiety OR (95% CI)
High HL group	Ref.	Ref.
Moderate HL group	5.16 (1.78–14.91) **	2.21 (1.09–4.46) *
Low HL group	8.06 (2.74–23.70) ***	3.01 (1.45–6.27) **

* *p* < 0.05, ** *p* < 0.01, *** *p* < 0.001; Ref.: Reference category

**Table 3:** The association of health literacy, depression and anxiety with diet non-adherence (Model 1) and the mediating effect (Model 2) of depression and anxiety in relation to HL and diet non-adherence. Logistic regression adjusted for age and gender (*n* = 479).

Characteristics	Model 1 OR (95% CI)	Model 2 OR (95% CI)
Health literacy		
High HL group	Ref.	Ref.
Moderate HL group	2.19 (1.21–3.99) **	1.97 (1.07–3.61) *^,1^
Low HL group	1.61 (0.85–3.06)	1.34 (0.70–2.60)
Depression		
No/low symptoms of depression	Ref.	Ref.
Moderate/severe symptoms of depression	1.94 (1.26–2.98) **	1.90 (1.22–2.96) **
Health literacy		
High HL group	Ref.	Ref.
Moderate HL group	2.19 (1.21–3.99) **	2.05 (1.12–3.76) *^,2^
Low HL group	1.61 (0.85–3.06)	1.44 (0.75–2.77)
Anxiety		
No/low symptoms of anxiety	Ref.	Ref.
Moderate/severe symptoms of anxiety	1.81 (1.22–2.69) **	1.77 (1.19–2.65) **

* *p* < 0.05, ** *p* < 0.01. The degree of reduction in the ORs: (OR (crude)—OR (adjusted)/(OR (crude)—1) × 100%; ^1^ 19.5%; ^2^ 11.8%; Ref.: Reference category.

## Appendix A

### Health Literacy Questionnaire—Detailed Description of the Measurement Tool

The Health Literacy Questionnaire (HLQ) consists of two parts. The first part is covering the domains 1–5 (feeling understood and supported by healthcare provider; having sufficient information to manage my health; actively managing my health; social support for health; appraisal of health information). The participants respond to the statements regarding health literacy skills and limitations on the Likert scale from 1 (strongly agree) to 4 (strongly disagree). The second part is covering the domains 6–9 (ability to actively engage with healthcare providers; navigating the healthcare system; ability to find good health information; understanding health information well enough to know what to do). The participants respond to the statements by choosing one of the five options reflecting their ability to perform certain tasks. These answering options are 1—Cannot do, 2—Very difficult, 3—Quite difficult, 4—Quite easy, 5—Very easy. The mean score for each domain is used for analysis. The domains 1–5 have a mean score ranging from 1 to 4; the domains 6–9 have a mean score ranging from 1 to 5.

## References

[1] Jha V., Garcia-Garcia G., Iseki K., Li Z., Naicker S., Plattner B., Saran R., Wang A.Y., Yang C.W. (2013). Chronic kidney disease: Global dimension and perspectives. Lancet.

[2] Hill N.R., Fatoba S.T., Oke J.L., Hirst J.A., O’Callaghan C.A., Lasserson D.S., Hobbs F.D. (2016). Global Prevalence of Chronic Kidney Disease—A Systematic Review and Meta-Analysis. PLoS ONE.

[3] Li H., Xie L., Yang J., Pang X. (2018). Symptom burden amongst patients suffering from end-stage renal disease and receiving dialysis: A literature review. Int. J. Nurs. Sci..

[4] Sørensen K., Van den Broucke S., Fullam J., Doyle G., Pelikan J., Slonska Z., Brand H., (HLS-EU) Consortium Health Literacy Project European (2012). Health literacy and public health: A systematic review and integration of definitions and models. BMC Public Health.

[5] Taylor D.M., Fraser S., Dudley C., Oniscu G.C., Tomson C., Ravanan R., Roderick P., ATTOM Investigators (2018). Health literacy and patient outcomes in chronic kidney disease: A systematic review. Nephrol. Dial. Transpl..

[6] Taylor D.M., Fraser S.D.S., Bradley J.A., Bradley C., Draper H., Metcalfe W., Oniscu G.C., Tomson C.R.V., Ravanan R., Roderick P.J. (2017). A Systematic Review of the Prevalence and Associations of Limited Health Literacy in CKD. Clin. J. Am. Soc. Nephrol..

[7] Cavanaugh K.L., Wingard R.L., Hakim R.M., Eden S., Shintani A., Wallston K.A., Huizinga M.M., Elasy T.A., Rothman R.L., Ikizler T.A. (2010). Low health literacy associates with increased mortality in ESRD. J. Am. Soc. Nephrol..

[8] Skoumalova I., Kolarcik P., Madarasova Geckova A., Rosenberger J., Majernikova M., Klein D., van Dijk J.P., Reijneveld S.A. (2019). Is Health Literacy of Dialyzed Patients Related to Their Adherence to Dietary and Fluid Intake Recommendations?. Int. J. Environ. Res. Public Health.

[9] Berkman N.D., Sheridan S.L., Donahue K.E., Halpern D.J., Viera A.J., Crotty K., Holland A., Brasure M., Lohr K.N., Harden E. (2011). Health Literacy Interventions and Outcomes: An Updated Systematic Review. Evid. Rep. Technol. Assess. (Full Rep.).

[10] Sheridan S.L., Halpern D.J., Viera A.J., Berkman N.D., Donahue K.E., Crotty K. (2011). Interventions for individuals with low health literacy: A systematic review. J. Health Commun..

[11] Ng H.J., Tan W.J., Mooppil N., Newman S., Griva K. (2015). Prevalence and patterns of depression and anxiety in hemodialysis patients: A 12-month prospective study on incident and prevalent populations. Br. J. Health Psychol..

[12] Kimmel P.L., Cukor D. (2019). Anxiety Symptoms in Patients Treated With Hemodialysis: Measurement and Meaning. Am. J. Kidney Dis..

[13] Watnick S., Kirwin P., Mahnensmith R., Concato J. (2003). The prevalence and treatment of depression among patients starting dialysis. Am. J. Kidney Dis..

[14] Kimmel P.L. (2002). Depression in patients with chronic renal disease: What we know and what we need to know. J. Psychosom. Res..

[15] Murtagh F.E., Addington-Hall J., Higginson I.J. (2007). The prevalence of symptoms in end-stage renal disease: A systematic review. Adv. Chronic Kidney Dis..

[16] Palmer S., Vecchio M., Craig J.C., Tonelli M., Johnson D.W., Nicolucci A., Pellegrini F., Saglimbene V., Logroscino G., Fishbane S. (2013). Prevalence of depression in chronic kidney disease: Systematic review and meta-analysis of observational studies. Kidney Int..

[17] Brito D.C.S., Machado E.L., Reis I.A., Carmo L.P.F.D., Cherchiglia M.L. (2019). Depression and anxiety among patients undergoing dialysis and kidney transplantation: A cross-sectional study. Sao Paulo Med. J..

[18] Fischer M.J., Kimmel P.L., Greene T., Gassman J.J., Wang X., Brooks D.H., Charleston J., Dowie D., Thornley-Brown D., Cooper L.A. (2010). Sociodemographic factors contribute to the depressive affect among African Americans with chronic kidney disease. Kidney Int..

[19] Bujang M.A., Musa R., Liu W.J., Chew T.F., Lim C.T., Morad Z. (2015). Depression, anxiety and stress among patients with dialysis and the association with quality of life. Asian J. Psychiatry.

[20] Ginieri-Coccossis M., Theofilou P., Synodinou C., Tomaras V., Soldatos C. (2008). Quality of life, mental health and health beliefs in haemodialysis and peritoneal dialysis patients: Investigating differences in early and later years of current treatment. BMC Nephrol..

[21] Farrokhi F., Abedi N., Beyene J., Kurdyak P., Jassal S.V. (2014). Association between depression and mortality in patients receiving long-term dialysis: A systematic review and meta-analysis. Am. J. Kidney Dis..

[22] Schouten R.W., Haverkamp G.L., Loosman W.L., Chandie Shaw P.K., van Ittersum F.J., Smets Y.F.C., Vleming L.J., Dekker F.W., Honig A., Siegert C.E.H. (2019). Anxiety symptoms, mortality and hospitalization in patients receiving maintenance dialysis: A cohort study. Am. J. Kidney Dis..

[23] Boulware L.E., Liu Y., Fink N.E., Coresh J., Ford D.E., Klag M.J., Powe N.R. (2006). Temporal relation among depression symptoms, cardiovascular disease events, and mortality in end-stage renal disease: Contribution of reverse causality. Clin. J. Am. Soc. Nephrol..

[24] Koo J.R., Yoon J.W., Kim S.G., Lee Y.K., Oh K.H., Kim G.H., Kim H.J., Chae D.W., Noh J.W., Lee S.K. (2003). Association of depression with malnutrition in chronic hemodialysis patients. Am. J. Kidney Dis..

[25] Weisbord S.D., Mor M.K., Sevick M.A., Shields A.M., Rollman B.L., Palevsky P.M., Arnold R.M., Green J.A., Fine M.J. (2014). Associations of depressive symptoms and pain with dialysis adherence, health resource utilization, and mortality in patients receiving chronic hemodialysis. Clin. J. Am. Soc. Nephrol..

[26] Cukor D., Peterson R.A., Cohen S.D., Kimmel P.L. (2006). Depression in end-stage renal disease hemodialysis patients. Nat. Clin. Pract. Nephrol..

[27] Palmer S.C., Vecchio M., Craig J.C., Tonelli M., Johnson D.W., Nicolucci A., Pellegrini F., Saglimbene V., Logroscino G., Hedayati S.S. (2013). Association between depression and death in people with CKD: A meta-analysis of cohort studies. Am. J. Kidney Dis..

[28] Lopes A.A., Bragg J., Young E., Goodkin D., Mapes D., Combe C., Piera L., Held P., Gillespie B., Port F.K. (2002). Depression as a predictor of mortality and hospitalization among hemodialysis patients in the United States and Europe. Kidney Int..

[29] McDade-Montez E.A., Christensen A.J., Cvengros J.A., Lawton W.J. (2006). The role of depression symptoms in dialysis withdrawal. Health Psychol..

[30] Khalil A.A., Frazier S.K., Lennie T.A., Sawaya B.P. (2011). Depressive symptoms and dietary adherence in patients with end-stage renal disease. J. Ren. Care..

[31] Cohen S.D., Cukor D., Kimmel P.L. (2016). Anxiety in patients treated with hemodialysis. Clin. J. Am. Soc. Nephrol..

[32] Qobadi M., Besharat M.A., Rostami R., Rahiminezhad A., Pourgholami M. (2015). Health literacy, negative emotional status, and self-care behaviors in dialysis. J. Fundam. Ment. Health.

[33] Stømer U.E., Gøransson L.G., Wahl A.K., Urstad K.H. (2019). A cross-sectional study of health literacy in patients with chronic kidney disease: Associations with demographic and clinical variables. Nurs. Open.

[34] Dodson S., Osicka T., Huang L., McMahon L.P., Roberts M.A. (2016). Multifaceted Assessment of Health Literacy in People Receiving Dialysis: Associations with Psychological Stress and Quality of Life. J. Health Commun..

[35] Green J.A., Mor M.K., Shields A.M., Sevick M.A., Palevsky P.M., Fine M.J., Arnold R.M., Weisbord S.D. (2011). Prevalence and demographic and clinical associations of health literacy in patients on maintenance hemodialysis. Clin. J. Am. Soc. Nephrol..

[36] (2013). KDIGO 2012 Clinical Practice Guideline for the Evaluation and Management of Chronic Kidney Disease. Kidney Int. Suppl..

[37] Lambert K., Mullan J., Mansfield K. (2017). An integrative review of the methodology and findings regarding dietary adherence in end stage kidney disease. BMC Nephrol..

[38] Kolarcik P., Cepova E., Madarasova Geckova A., Elsworth G.R., Batterham R.W., Osborne R.H. (2017). Structural properties and psychometric improvements of the Health Literacy Questionnaire in a Slovak population. Int. J. Public Health.

[39] Osborne R.H., Batterham R.W., Elsworth G.R., Hawkins M., Buchbinder R. (2013). The grounded psychometric development and initial validation of the Health Literacy Questionnaire (HLQ). BMC Public Health.

[40] Dempster A.P., Laird N.M., Rubin D.B. (1997). Maximum Likelihood from Incomplete Data via the EM Algorithm. J. R. Stat. Soc. Ser. B..

[41] Beauchamp A., Buchbinder R., Dodson S., Batterham R.W., Elsworth G.R., McPhee C., Sparkes L., Hawkins M., Osborne R.H. (2015). Distribution of health literacy strengths and weaknesses across socio-demographic groups: A cross-sectional survey using the Health Literacy Questionnaire (HLQ). BMC Public Health..

[42] Ward J.H. (1963). Hierarchical Grouping to Optimize an Objective Function. J. Am. Stat. Assoc..

[43] Zigmond A., Snaith R. (1983). The hospital anxiety and depression scale. Acta Psychiatr. Scand..

[44] Martin C.R., Tweed A.E., Metcalfe M.S. (2004). A psychometric evaluation of the Hospital Anxiety and Depression Scale in patients diagnosed with end-stage renal disease. Br. J. Clin. Psychol..

[45] Loosman W.L., Siegert C.E.H., Korzec A., Honig A. (2010). Validity of the Hospital Anxiety and Depression Scale and the Beck Depression Inventory for use in end-stage renal disease patients. Br. J. Clin. Psychol..

[46] Bjelland I., Dahl A.A., Haug T.T., Neckelmann D. (2002). The validity of the Hospital Anxiety and Depression Scale. An updated literature review. J. Psychosom. Res..

[47] Yoong R.K., Mooppil N., Khoo E.Y., Newman S.P., Lee V.Y., Kang A.W., Griva K. (2017). Prevalence and determinants of anxiety and depression in end stage renal disease (ESRD). A comparison between ESRD patients with and without coexisting diabetes mellitus. J. Psychosom. Res..

[48] Olagunju A.T., Campbell E.A., Adeyemi J.D. (2015). Interplay of anxiety and depression with quality of life in endstage renal disease. Psychosomatics.

[49] Stømer E.U., Wahl K.A., Gøransson G.L., Urstad H.K. (2020). Health literacy in kidney disease: Associations with quality of life and adherence. J. Ren. Care.

[50] Lin C.Y., Ganji M., Griffiths M.D., Bravell M.E., Broström A., Pakpour A.H. (2019). Mediated effects of insomnia, psychological distress and medication adherence in the association of eHealth literacy and cardiac events among Iranian older patients with heart failure: A longitudinal study. Eur. J. Cardiovasc. Nurs..

